# Suspicious Behavior Detection with Temporal Feature Extraction and Time-Series Classification for Shoplifting Crime Prevention

**DOI:** 10.3390/s23135811

**Published:** 2023-06-22

**Authors:** Amril Nazir, Rohan Mitra, Hana Sulieman, Firuz Kamalov

**Affiliations:** 1 College of Technological Innovation, Zayed University, Abu Dhabi, United Arab Emirates; 2 Department of Computer Science and Engineering, American University of Sharjah, Sharjah, United Arab Emirates; b00085023@aus.edu; 3 Department of Mathematics and Statistics, American University of Sharjah, Sharjah, United Arab Emirates; hsulieman@aus.edu; 4 Department of Electrical Engineering, Canadian University Dubai, Dubai, United Arab Emirates

**Keywords:** automated crime detection, suspicious behavior, crime prevention, temporal features, time-series classification

## Abstract

The rise in crime rates in many parts of the world, coupled with advancements in computer vision, has increased the need for automated crime detection services. To address this issue, we propose a new approach for detecting suspicious behavior as a means of preventing shoplifting. Existing methods are based on the use of convolutional neural networks that rely on extracting spatial features from pixel values. In contrast, our proposed method employs object detection based on YOLOv5 with Deep Sort to track people through a video, using the resulting bounding box coordinates as temporal features. The extracted temporal features are then modeled as a time-series classification problem. The proposed method was tested on the popular UCF Crime dataset, and benchmarked against the current state-of-the-art robust temporal feature magnitude (RTFM) method, which relies on the Inflated 3D ConvNet (I3D) preprocessing method. Our results demonstrate an impressive 8.45-fold increase in detection inference speed compared to the state-of-the-art RTFM, along with an F1 score of 92%,outperforming RTFM by 3%. Furthermore, our method achieved these results without requiring expensive data augmentation or image feature extraction.

## 1. Introduction

The problem of suspicious-behavior detection is crucial in addressing crime prevention. Despite a myriad of work related to anomaly detection in videos, there has been little focus on suspicious-behavior detection tasks for crime prevention. Crime prevention detection is a very difficult task due to several reasons. First, crime prevention requires detecting suspicious behavior before the actual crime occurs. This is a considerable challenge, since typical suspicious behavior in a slow-paced environment is highly similar to normal behavior. Second, abnormal actions from suspicious activities generally occur at a slower pace compared to actual criminal activities such as theft, burglary, and robbery. Third, it often requires relatively longer observations of a person’s activity before any inference can be made. Lastly, there are significantly fewer data available for training the model. In this project, we propose an effective method for detecting suspicious behavior that works with minimal training.

With the advancement of computer vision technology, various techniques have been proposed for crime prevention through video-anomaly detection. However, the effectiveness of these techniques varies depending on the use cases, such as traffic monitoring, crowd monitoring, and security surveillance. In this paper, we specifically focus on the use case of retail theft prevention, which aims to identify suspicious behavior in retail stores such as shoplifting or similar types of theft.

Several state-of-the-art techniques for preventing shoplifting have been proposed, including those by Kirichenko L. et al. [1], Gandapur et al. [2], Qin Z. et al. [3], and Wu Y. et al. [4]. These techniques heavily rely on CNN-based feature extraction methods that use pretrained models to extract visual appearance and optical flow features in order to represent spatiotemporal information. However, such methods are computationally costly, and prone to excessive and redundant information. For example, Inflated 3D ConvNet (I3D) is the state-of-the-art deep learning preprocessing method for video classification and action recognition in computer vision. It was proposed by researchers at Google in 2017, and it builds on the success of 2D convolutional neural networks (CNNs) by extending them to 3D. I3D uses 3D convolutional layers to process spatiotemporal data in video frames. It starts with a pretrained 2D CNN on ImageNet, and then extends it to 3D by inflating each 2D filter into a 3D filter. This allows for the network to capture both spatial and temporal information from video frames. The I3D achieved state-of-the-art results on several benchmark datasets for action recognition, including Kinetics and HMDB51. It is widely used in research and applications related to security surveillance.

The I3D method is a powerful approach to video analysis, but it demands a considerable amount of processing power to preprocess each frame that can take around 0.5 to 5 s to preprocess a single frame using I3D on a high-spec hardware configuration. Object detection techniques such as YOLO, Faster R-CNN, and Mask R-CNN, on the other hand, can perform real-time video processing, detecting and extracting human object bounding box coordinates at a rate of 30 frames per second or higher. Thus, by efficiently utilizing video frame features in real time to detect suspicious behavior (without relying on I3D or similar approach), it is possible to significantly reduce the inference detection time.

The question that arises next is which features we should extract from video frames to effectively detect suspicious behaviors. Our hypothesis is that, by examining a sequence of video frames depicting a person’s activity and behavior, we can increase the likelihood of detecting suspicious behavior. Time-series deep learning classification models can be trained to track and learn the sequences of people’s actions and movements, which can lead to more efficient and improved detection performance. To achieve this, we propose a novel approach that involves extracting the bounding box features of individuals in CCTV videos, and analyzing the data using time-series deep learning algorithms. We aim to address the following research questions:

- How effective is using a sequence of video frames depicting a person’s activity and behavior in increasing the likelihood of detecting suspicious behavior?
- How can time-series deep learning classification models be trained to track and learn sequences of individual actions and movements to improve detection performance?
- How does the proposed method compare to the I3D preprocessing method and the state-of-the-art Robust Temporal Feature Magnitude (RTFM) deep learning anomaly detection method in terms of detection performance on shoplifting incidents?

To this end, we propose YOLOv5 with Deep Sort method to detect and track individuals across multiple frames in video sequences. We then extract the resulting bounding box coordinates as temporal features and use them as inputs to time-series classification deep learning models. We evaluated our approach using the UCF Crime dataset, which includes labeled video frames of shoplifting incidents. Our proposed method was compared against the state-of-the-art Robust Temporal Feature Magnitude (RTFM) deep learning anomaly detection method. Our results revealed that our method exhibits faster detection speed and higher accuracy scores. Impressively, we achieved an 8.45-fold increase in detection speed and a F1 score of 92%, surpassing RTFM by 3%, all without the need for data augmentation or I3D image feature extraction.

The paper is organized as follows: Section 2 provides a review of the related work, while Section 3 details the proposed method. Section 4 describes the used datasets and the applied preprocessing techniques. The experimental setup is discussed in Section 5, while Section 6 presents the results and their discussion. Lastly, in Section 7, the paper is concluded with a summary of the findings.

## 2. Related Work

Anomaly detection has been widely studied in computer vision [5,6,7,8,9] in various problem settings such as fighting/violence alerts, people fall detection, unusual pedestrian motion patterns, and traffic accidents. Three common techniques have been extensively studied in the field of video anomaly detection, namely, unsupervised [10,11,12,13,14,15], supervised [16,17,18], and semi-supervised/weakly supervised [19,20,21,22,23,24]. The unsupervised technique attempts to detect abnormal activities where no labeled normal/abnormal training data are provided. The supervised technique, on the other hand, uses labeled normal/anomalous data during the training process. Recently, the semi-supervised/weakly supervised technique has been discovered, and it is often cited as the state-of-the-art technique for video anomaly detection. The weakly supervised technique uses video-level labels to selectively segment videos into normal and abnormal frames. During the training phase, if a video comprises all normal events, each frame is labeled as normal. However, if the video has at least one abnormal frame, all the frames in the video (including the normal event frames) are labeled as abnormal. This labeling assignment is known as “noisy labels” in the literature because normal frames are labeled as abnormal. After such labeling, a rank loss is applied to assign higher scores to the anomaly frame in the video. Recent results [19,20] showed that these approaches are very effective in video anomaly detection.

Due to the extensive work of video anomaly detection, it is not feasible or practical to compare the effectiveness of different techniques without considering the use cases. In this paper, we specifically focus on shoplifting crime prevention. The objective is to detect a crime via the suspicious behavior of individuals before the actual shoplifting crime occurs.

Kirichenko L. et al. [2] presented a hybrid neural network for detecting shoplifting in video records. The network combined convolutional and recurrent networks, with gated recurrent units being used as the recurrent component. Ansari et al. [25] proposed a dual-stream convolutional neural network and a long short-term memory (LSTM)-based deep learner to extract appearance and salient motion features from video sequences.

Gandapur et al. [2] proposed a three-layered bidirectional gate recurrent unit (BiGRU) and a convolutional neural network (CNN). The CNN was used to extract the spatial features from video frames, whereas temporal and local motion features were extracted by the BiGRU from the CNN-extracted features of multiple frames. The limitation of this method is due to the video frames of certain actions that need to be manually chosen as part of the video-processing phase. The approach also requires ranked-based loss to effectively detect and classify suspicious activity.

Qin Z. et al. [3] proposed a two-stage method to detect and prevent criminal activities in shopping malls in real time. The first stage involves the CNN feature extraction method using a pretrained VGG-16 model. The second involves a classification task using either SVM or LSTM using a custom ranking loss.

Wu Y. et al. [4] proposed a three-dimensional convolutional neural network (3D-CNN) to extract information features from continuous multiframe cube data and acquire the features of spatial–temporal dimensions. A three-dimensional CNN was proposed to represent time-series information on continuous multiframe cube data. The input data of the three-dimensional CNN were cube data composed of multiple consecutive video frames aiming to improve crime detection events.

These state-of-the-art techniques rely heavily on CNN-based feature extraction methods (e.g., I3D, S3D, CLIP, RAFT, ResNet, and VGGish) using a pretrained model to extract visual appearance and optical flow features in order to represent spatiotemporal information. The learned feature representations from such methods are susceptible to excessive and redundant information because they track all surfaces and edges on each frame. Furthermore, they are computationally costly because each object and scene typically move in each video frame. In this paper, we propose a much simpler and more efficient approach to capturing and extracting spatiotemporal features. Rather than capturing all motion objects and surfaces, we aimed to capture the sequence of the bounding boxes of individuals at consecutive frames. This minimizes the motion-feature representation into four attributes (i.e., x1, x2, y1, y2) for each person. The movement velocity of each person is also tracked and represented as a sequence of time-series data points. This approach has significantly fewer motion-feature representations and less computational overhead compared to those of existing approaches.

## 3. Proposed Method

The proposed approach is based on a two-stage algorithm consisting of feature extraction and deep-learning time-series analysis. The first stage uses YOLOv5 with Deep Sort to track individuals across a video and capture the bounding box information that tracks each individual in the video. The extracted information from the bounding boxes is used to create a new dataset. In the second stage, the extracted temporal features are supplied to deep-learning models for analysis and classification.

**Figure 1:**
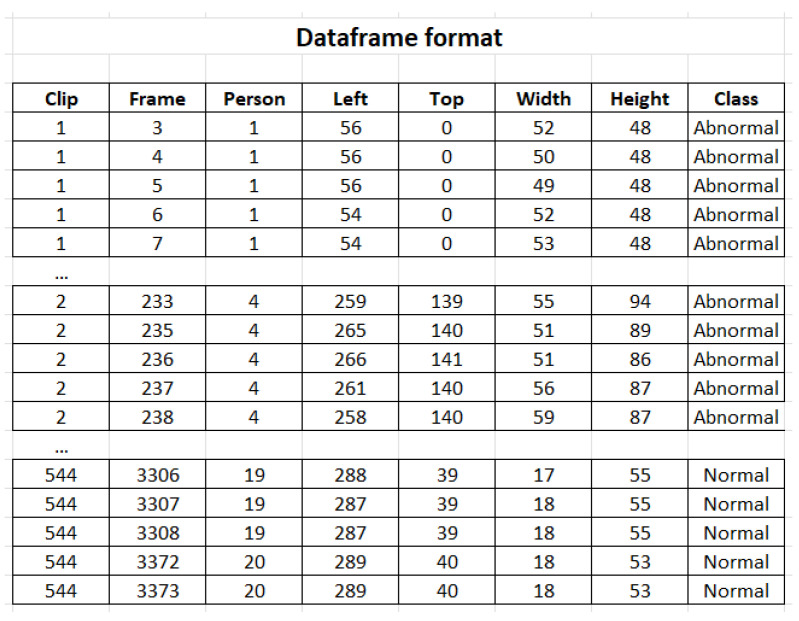
Extracted dataset format.

The first stage employs object detection and video tracking algorithm YOLOv5 with Deep Sort [26] to track multiple individuals across each video. This allows for the extraction of bounding box features across multiple time steps. Hence, the video-based dataset is converted into a tabular format, as shown in Figure 1.

The second stage of the proposed method involves applying various state-of-the-art time-series deep-learning methods to the extracted dataset. We explored different time-series classification algorithms imported from the popular Time Series Artificial Intelligence (TSAI) library.

**Figure 2:**
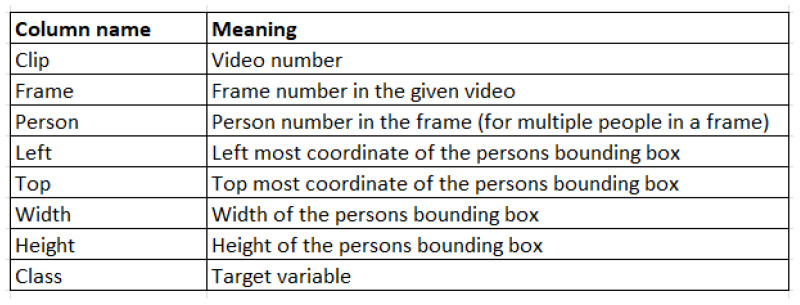
Description of the columns in Figure 1.

The final dataset is constructed by noting the video number, the frame number, and the bounding box coordinates of each person in the frame as identified by YOLOv5 with Deep Sort. An example of the final dataset structure is shown in Figure 1. The description of the columns in the dataset is provided in Figure 2. This conversion of video data into a tabular format using YOLOv5 with Deep Sort leads to rapid data processing and includes no other preprocessing or particular image data augmentation techniques, allowing for quick data augmentation into the described tabular format.

We employed YOLOv5 with Deep Sort using the default parameters loaded with the pretrained weights from crowdhuman_yolov5m [26]. This set of weights were chosen because they were trained to identify humans in crowded scenarios, which applies to our case. Moreover, we configured the model to only track humans and not any other animals/objects by setting the class parameter to 0. Hence, all the resulting bounding boxes tracked people through multiple frames.

**Figure 3:**
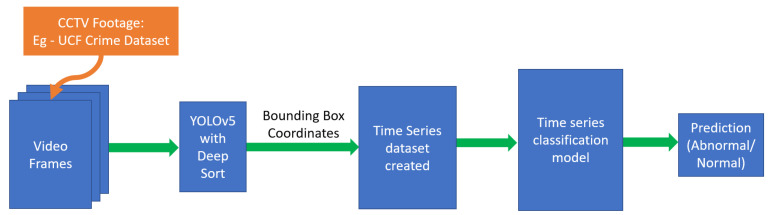
Proposed model pipeline.

The proposed method for tracking people through videos reduces the impact of cluttered videos, and removes any possible dependency of the classification model on the scene itself, which allows for better generalization. Instead, a popular model such as YOLOv5 with Deep Sort is able to easily identify humans in different scenarios and focus on only that, while classification models focus more on analyzing the movement and positions of people. The proposed pipeline is illustrated in Figure 3.

**Figure 4:**
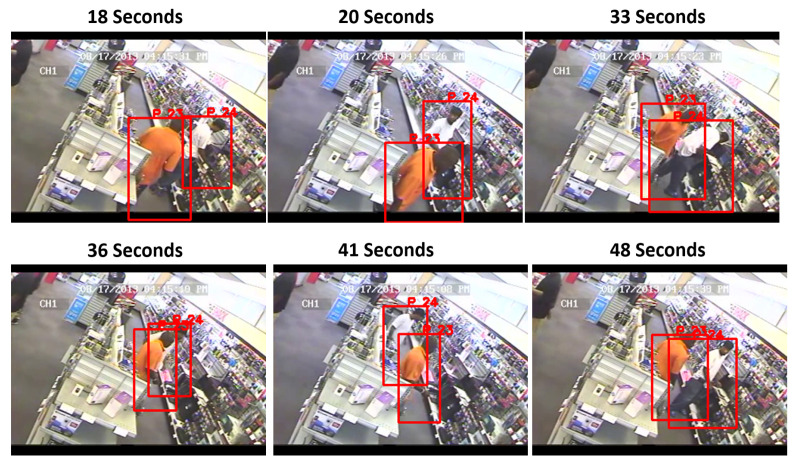
Capturing bounding boxes with YOLOv5 with Deep Sort.

An example of the bounding boxes tracking people in successive frames can be seen below. Examining the video at 18, 20, 33, 36, 41, and 48 s in Figure 4 demonstrates how Deep Sort was able to track two individuals in a store and consistently label each person as P23 and P24 (Persons Number 23 and 24). This implementation successfully allowed for tracking multiple people, even if they switched positions, as was the case at 41 and 48 s, when the people switched positions, but the algorithm was able to consistently track both of them.

Existing state-of-the-art anomaly detection models, such as RTFM, apply sophisticated data transformation and segmentation techniques to train deep-learning models. In contrast, instead of using pixels as features (as in CNN-based models), our approach generates the numerical features of human activity that represent coordinates from a real-time person tracker. A real-time person tracker generates movement coordinates for each time frame that could be used as features for our deep learning model.

Due to the nature of the time-series data from the coordinates and time frame, we employed recurrent neural networks capable of learning the order in sequence prediction problems. Therefore, long short-term memory (LSTM)-based networks are proposed as the ideal candidate for Stage 2 of the proposed approach due to their ability to capture temporal dependencies. We also explored state-of-the-art time-series deep learning classification models An Explainable Convolutional Neural Network (Fauvel, 2021) and MiniRocket (Dempster, 2021), as they both offer the best performance for time-series classification tasks.

## 4. Data

### 4.1. Datasets

The used dataset in the numerical experiments was the popular UCF Crime dataset [27] that contains approximately 128 h of video and 2 types of data—normal and crime videos. The crime-labeled samples consisted of videos capturing the occurrence of different illegal activities, including crimes such as abuse, arrest, arson, assault, road accidents, burglary, explosion, fighting, robbery, shooting, stealing, shoplifting, and vandalism. In our study, we considered shoplifting crimes, as they include the most suspicious behavior before the theft occurs. However, the proposed method is widely applicable to a series of video-based anomaly detection problems where the pattern of movement of some objects is what constitutes an anomaly. For example, the proposed approach would also work to detect anomalies such as loitering, traffic rule violations, and stampedes.

In particular, the UCF dataset contains videos of people shopping in different shops. Normal class videos are people who are browsing items in different shops. Abnormal class videos contain videos of people who are stealing from each shop. After we had selected the shoplifting instances described above, the dataset contained 267 normal class videos and 50 abnormal class videos. While there was a class imbalance in the data, the bounding box data extracted from the videos were large enough to eliminate any significant classification bias during model training.

### 4.2. Dataset Preprocessing

The first stage of the proposed method employs object detection and video tracking algorithm YOLOv5 with Deep Sort to track multiple people across each video. This allows for the extraction of bounding box features across multiple time steps to create a tabular dataset.

Upon extracting the bounding box features, the resulting dataset (Figure 1) contained more rows than the original frames did because a frame could have multiple identified people, which caused the number of rows in each frame to increase.

To manage the computational load, each video that contained over 15,000 rows in the tabular dataset was split into multiple clips, with each clip having at most 15,000 rows. We lastly obtained 544 clips from the 317 original videos. The updated distribution of the class labels is presented in Table 1. The percentage of abnormal instances in the dataset was 14.92%.

In our experiment, we split the data into training and test subsets using a stratified split as shown in Table 2. We maintained the overall percentage of abnormal instances after the split to 14.93%.

## 5. Experimental Setup

### 5.1. Time-Series Deep-Learning Classification Models

We began by running YOLOv5 with Deep Sort on the UCF Crime dataset, and converting the videos into the format described in Figure 1. Once the dataset had been constructed, we explored popular models from the TSAI library to perform time-series classification on the tabular dataset. The dataset was split as described in Table 2.

The following TSAI models are considered in our study: InceptionTime [28], XceptionTime [29], Explainable Convolutional Network (XCM) [30], and MiniRocket [31]. InceptionTime and XceptionTime are time-series classification models that were adapted from popular convolutional neural network models Inception and Xception, respectively, which are often used for image classification. On the other hand, XCM and MiniRocket are more recent time-series classification models. Each model was trained on the tabular data generated by YOLOv5 with Deep Sort for 15 epochs; afterwards, the model was saved and tested.

#### 5.1.1. InceptionTime

The InceptionTime architecture was inspired by the inception convolutional neural network, where an inception network is built from inception modules for multivariate time-series classification. Each inception module has a bottleneck layer that simply performs the operation of sliding a convolutional filter with length 1 and stride 1 along the time series to reduce its dimensionality. This helps in converting a multivariate time series into a time series with fewer independent variables, and improving model complexity, which helps in avoiding overfitting for small datasets. The use of the bottleneck layer allows for the inception module to have longer filters while maintaining the same number of parameters as that of ResNet, allowing for it to capture a larger range of data from which to learn.

**Figure 5:**
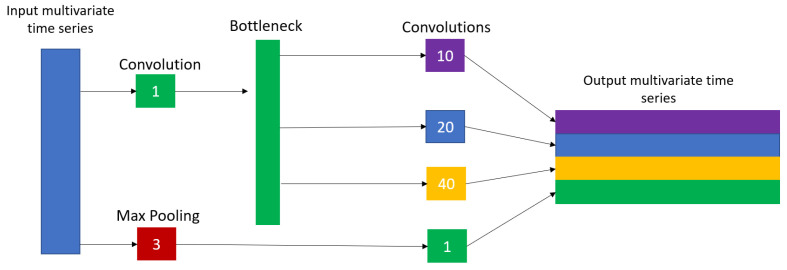
Inception module proposed in [28].

Another key feature of the inception module is that it convolves multiple filters of varying sizes (sizes 10, 20, and 40, as shown in Figure 5) in parallel to the output of the bottleneck layer. In addition, the model performs max pooling on the input time series over a fixed window size by simply taking the maximal value of the time series within that window to ensure that the module is robust and not affected by noise. Lastly, the module concatenates the results of the varying-size convolutions with the max pooling bottleneck to construct the output time series of this module.

**Figure 6:**
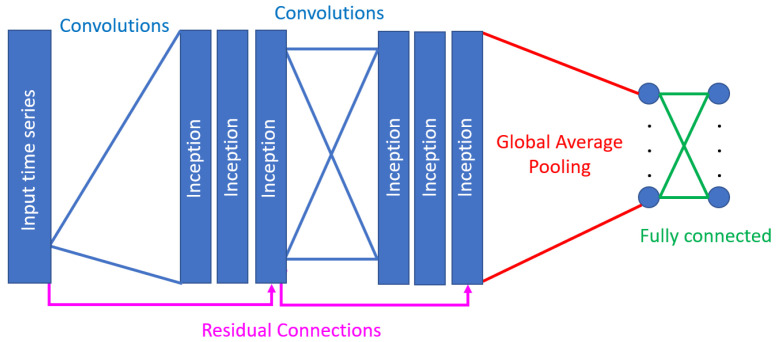
Inception network proposed in [28].

An inception network, as shown in Figure 6, is built using inception modules in 2 residual blocks of 3 modules each. Each residual block’s input comes directly from a linear connection from the previous block’s output, while there are also convolutions between successive inception modules to ensure that it is able to capture trends from within the time series. This use of residual connections to bypass convolutions is to ensure that the model does not suffer from vanishing gradients. The output of the final block of modules is then passed through global average pooling over the entire time dimension of the input, feeding the pooled time series into a fully connected neural network with dense layers and softmax activation for the final classification.

The InceptionTime classifier is based on an ensemble of five inception networks with multiple branches of convolutions and varying dilation rates, which allows for the model to capture both long- and short-term trends in the data. Ensembling is conducted using the following function:

(1) $${\hat{y}}_{i,c}=\frac{1}{n}\sum_{j=1}^{n}\sigma_{c}\left(x_{i},\theta_{j}\right),\forall c\in \left[1,C\right],$$

where ${\hat{y}}_{i,c}$ denote the ensemble’s output probability of having input time series $x_{i}$ belonging to class *c*, which is equal to the logistic output $\sigma_{c}$ averaged over *n* randomly initialized models. Stacking inception networks and using backpropagation allow for the model to learn the latent hierarchical features of multiple resolutions through the time series.

#### 5.1.2. XceptionTime

**Figure 7:**
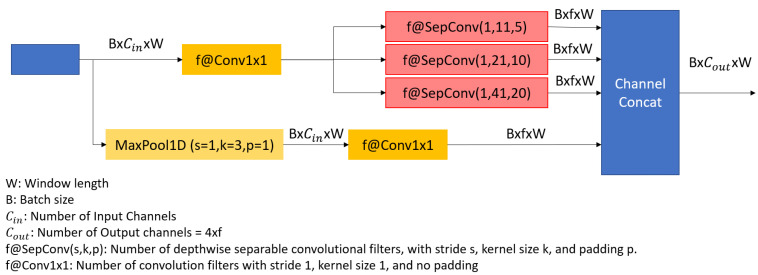
XceptionTime module proposed in [29].

**Figure 8:**
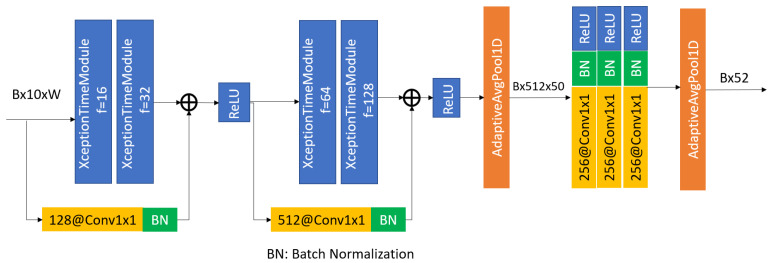
Overall XceptionTime architecture proposed in [29].

The novel XceptionTime architecture was inspired by Inception and AlexNet, proposed in [29]. The authors developed the XceptionTime module shown in Figure 7. This model passes the input data through two routes, one with three depthwise separable convolutions, and another through max pooling and a singleton convolution. These XceptionTime modules are used to build the final time-series classifier, as shown in Figure 8, which contains two pairs of XceptionTime modules with residual connections, followed by adaptive average pooling and singleton convolutions.

A single XceptionTime module, as shown in Figure 7, consists of a bottleneck layer that uses a single convolutional filter with stride 1 and kernel size 1 to reduce the dimensionality of the input time series and potential issues with overfitting. Moreover, it uses the idea of simultaneously passing multiple filters with varying kernel sizes over the output of the bottleneck layer. However, unlike InceptionTime modules, depthwise separable convolutions are used here. Depthwise separable convolutions are split into two types: depthwise and pointwise. Depthwise convolutions perform each convolution separately for each input channel and then stacked together. Subsequently, the output from depthwise convolution is passed into the pointwise convolutions of 1 × 1 convolutions to transform the data into having smaller channel depth. This helps in reducing the number of parameters to train the model, and improves training time. The output of multiple simultaneous depthwise separable convolutions and the output of a max pooling with a 1 × 1 convolution are concatenated along their channels to produce the final output time series.

These XceptionTime modules are used in the overall architecture of the XceptionTime classifier by stacking two pairs of modules together with different filter sizes, as shown in Figure 8. Moreover, XceptionTime consists of residual connections around each block of the XceptionTime modules to ensure that there are no vanishing gradients during training. The output of the last XceptionTime module is then fed into an adaptive average pooling layer, followed by a set of convolutional layers to match the input and output dimensions, and then batch normalization to reduce the internal covariate shift effect.

#### 5.1.3. MiniRocket

MINImally RandOm Convolutional KErnel Transform (MiniRocket) is an improvement on ROCKET to eliminate randomness and render it almost deterministic, and speed up the transformation processes, which is part of the original ROCKET model. The heart of the ROCKET and miniRocket classifiers is a transform to the input data that provides new features and is fed into a linear classifier such as logistic regression.

Moreover, the model is rendered more deterministic by fixing the kernel length to 9 for all applications. Furthermore, it restricts the weight values to a combination of two parameters: α, which is set to −1, and β, which is set to 2. The kernel is a permutation of α and β of length 9. However, there is a restriction: the overall sum of the weights should be zero. Additionally, biases are determined on the basis of the convolutional output. These biases are picked from quantiles of the convolutional output from a randomly chosen data point. This random choice of a training instance is the only nondeterministic process in the model. Lastly, the dilation of the convolutional kernels was fixed for each input and was chosen from the set $D=\{\left\lfloor 2^{0}\right\rfloor ,\cdots ,\left\lfloor 2^{max}\right\rfloor \}$, where the exponents were uniformly distributed between 0 and $max=log_{2}\left(\left(l_{input}-1\right)/\left(l_{kernel}-1\right)\right)$. Here, $l_{kernel}$ is the length of kernel (9), and $l_{input}$ is the length of the input time series. This range was chosen such that the maximal effective length of a kernel with dilation was equal to the length of the input time series. To avoid having dilations that are too large and reduce the number of extracted features, the dilation size was capped at 32. The improvement in speed comes from parallelization, reusing precomputed convolutions, avoiding certain multiplications, and computing multiple kernels at once.

The model computes the positive predictive value (PPV) for kernel *W* and mathematically transforms it to obtain its negation, −W. This requires the model to only compute a single kernel for the update rather than two matrices, almost doubling the speed. Moreover, the model performs a convolution on a single kernel (with fixed dilation) and reuses the output to compute multiple features by varying the bias values. This further reduces the number of operations to be performed. Additionally, the kernel weights being restricted to only two values (α and β) allows for us to mathematically take the multiplication term out as a common factor during computation of the convolution. This leads to the convolutional operation being only an addition with a single multiplication at the end. This speeds up the process, since matrix multiplication is an expensive operation. Lastly, since the kernel weights are restricted to two values, we could compute all the kernels in fewer operations (one for each value of the kernel weight). The kernel is computed on the basis of the values of α and β, and then adjusted on the basis of the used dilation value. This is the last step in optimizing the transform performed by ROCKET.

#### 5.1.4. Explainable Convolutional Network (XCM)

The Explainable Convolutional Network (XCM) is a novel architecture proposed for multivariate time-series classification. The main purpose of the architecture is to provide a certain level of explainability if required using explainable AI method GradCAM [32] by directly extracting related information to the observed variables and time from the input data by simultaneously applying 1D and 2D convolutions to the input data. Moreover, it passes the outputs of the parallel convolutions through a 1D global average pooling layer before passing it to a softmax layer for final classification. This reduces the number of parameters and improves the ability of the model to generalize while providing robustness to the spatial translations of the input. Lastly, the convolutional layers are fully padded, which allows for methods such as gradient-weighted class activation mapping (GradCAM) to be reliably used on the model. We were more interested in the ability of our application to outperform the SOTA methods in multivariate time-series classification methods.

**Figure 9:**
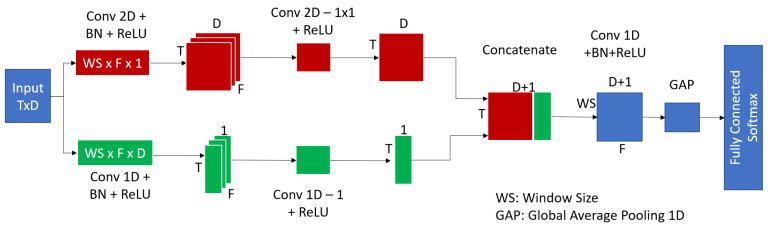
XCM architecture proposed in [30].

The proposed architecture can be seen in Figure 9. The architecture begins by using 2D convolutional filters to extract information about each of the observed variables, and using 1D convolution filters to extract temporal information. The use of both 1D and 2D convolutions leads to the extraction of more discriminative features by incorporating all the relevant information (both spatial and temporal) as compared to similar methods that use only 2D convolutions and fail to accurately capture temporal information. The window size for these filters can change as a hyperparameter to the model. The output from each convolutional block is passed through a ReLU layer to increase the generalization of the model, and through a 1 × 1 convolutional filter to project the feature maps onto channelwise pooling. The outputs of these two sections are concatenated and passed through a 1D convolutional layer and global average pooling layer that operate along the time axis to better capture interfeature interaction. Lastly, the output of the global average pooling layer is passed through dense layers with softmax activation for classification.

### 5.2. Baseline Comparison: Robust Temporal Feature Magnitude (RTFM)

We compared the proposed approach with the state-of-the-art robust temporal feature magnitude (RTFM) model [33], which is commonly applied to anomaly detection in videos using the same dataset. Therefore, it is a relevant and directly comparable benchmark to our method.

The RTFM model is based on defining a novel loss function that is dependent on the joint optimization of an end-to-end multiscale temporal feature learning and feature magnitude learning and an RTFM-enabled MIL classifier. The proposed function is as follows:

(2) $$\min_{\theta ,\phi }\sum_{i,j=1}^{\left|D\right|}{ł}_{s}\left(s_{\theta }\left(F_{i}\right),s_{\theta }\left(F_{j}\right),y_{i},y_{j}\right)+{ł}_{f}\left(f_{\phi }\left(s_{\theta }\left(F_{i}\right)\right),y_{i}\right),$$

where $s_{\theta }:F\to \chi$ is the temporal feature extractor (with $\chi \subset R^{TxD}$), $f_{\phi }:\chi \to {\left[0,1\right]}^{T}$ is the snippet classifier, $l_{s}$ denotes a loss function that maximizes the separability between the top-k snippet features from normal and abnormal videos, and $l_{f}$ is a loss function to train snippet classifier $f_{\phi }$ by also using the top-k snippet features from normal and abnormal videos.

Here, ${ł}_{f}$ is inspired by binary cross entropy and is defined as follows:

(3) $${ł}_{f}\left(f_{\phi }\left(s_{\theta }\left(F\right)\right),y\right)=\sum_{x\in \Omega_{k}\left(X\right)}-\left(y\log\left(f_{\phi }\left(X\right)\right)\right)+\left(1-y\right)\log\left(1-f_{\phi }\left(X\right)\right)$$

where $x=s_{\theta }\left(f\right)$. Moreover, to accurately model $s_{\theta }$, we must first define a few optimization functions. Initially, an optimization based on the mean feature magnitude of the top *k* snippets from a video:

(4) $$g_{\theta ,k}\left(X\right)=\max_{\Omega_{k}\left(X\right)\subseteq {\left\{x_{t}\right\}}_{t=1}^{T}}1/k\sum_{x_{t}\in \Omega_{k}\left(X\right)}\left|\right|x_{t}{\left|\right|}_{2}$$

where $g_{\Omega ,k}\left(.\right)$ is parameterized by θ, which allows for it to produce $x_{t}$. $\Omega_{k}\left(X\right)$ contains a subset of *k* snippets from ${x_{t}}_{t=1}^{T}$ and $|\Omega_{k}\left(X\right)|=k$. Next, the separability between normal and abnormal videos is denoted by the following:

(5) $$d_{\theta ,k}\left(X^{+},X^{-}\right)=g_{\theta ,k}\left(X^{+}\right)-g_{\theta ,k}\left(X^{-}\right)$$

where $X^{+}$ and $X^{-}$ represent the positive and negative class instances, respectively.

Using the above two equations, we define a loss function to model function $s_{\theta }\left(F\right)$ so that it minimizes the top *k* largest snippet feature magnitudes of normal videos and maximizes the same for abnormal videos.

(6) $${ł}_{s}\left(s_{\theta }\left(F_{i}\right),s_{\theta }\left(F_{j}\right),y_{i},y_{j}\right)=\left\{\begin{array}{cc} max(0,m-d_{\theta ,k}\left(X_{i},X_{j}\right)) & \mathrm{if}\;y_{i}=1,y_{j}=0\\ 0 & otherwise \end{array}\right.$$

where *m* is a predefined margin, $X_{i}=s_{\theta }\left(F_{i}\right)$ is the abnormal video feature, $X_{j}$ is the normal video feature, and $d_{\theta ,k}\;\mathrm{and}\;g_{\theta ,k}$ are defined as above. The proposed model was trained on the basis of the above loss function, which ensured the usage of inherent feature magnitude learning.

In addition, RTFM uses a preprocessing technique for video known as I3D features that involves passing each frame through ResNet50 and saving the encoded vectors obtained after passing it through all the convolutional layers. Hence, each video was converted into its corresponding I3D encoding before providing it as input to RTFM.

The split dataset used to train and test RTFM is shown in Table 3. Once again, to ensure our results were still comparable, we ensured that the percentage of abnormal instances was close to the original 14.9%; in this case, it was 15%. RTFM was trained with the default settings over 15,000 epochs, monitoring the metrics every 100 epochs.

Since none of the clips was inherently longer than 15,000 frames, no cropping was necessary. The proposed method had more instances in its dataset because any videos with over 15,000 rows were split into multiple videos. However, the methods remained comparable, since the overall percentage of abnormal instances remained the same—≈15%. Moreover, the original dataset used here was the same as that used for RTFM, and our proposed method allowed for us to maintain a level of comparability between approaches.

### 5.3. Evaluation Metrics

We track five primary metrics across this paper: accuracy, precision, recall, F1 score, and AUC. To convert the obtained probabilities by the model into the two classes, we used a threshold of 0.5 (i.e., the instance was classified as Class 1 (anomaly) if the probability for the class was ≥0.5; otherwise, it was Class 0 (normal)).

The used metrics were particularly useful, since the dataset was imbalanced. The first four metrics were derived on the basis of the confusion matrix using true-positive (TP), true-negative (TN), false-positive (FP), and false-negative (FN) values as follows:

(7) $$\mathrm{Accuracy}=\frac{\mathrm{TP}+\mathrm{TN}}{\mathrm{TP}+\mathrm{FP}+\mathrm{FN}+\mathrm{TN}},$$

(8) $$\mathrm{Precision}=\frac{\mathrm{TP}}{\mathrm{TP}+\mathrm{FP}},$$

(9) $$\mathrm{Recall}=\frac{\mathrm{TP}}{\mathrm{TP}+\mathrm{FN}},$$

(10) $$F1=\frac{2*\mathrm{Recall}*\mathrm{Precision}}{\mathrm{Recall}+\mathrm{Precision}},$$

The area under receiver operating characteristic curve (AUC) was calculated by plotting the true positive rate (TPR) and false positive rate (FPR) models. This is a probability curve, while the area under that curve represents the degree of the separability of the two classes. This tells us how capable the model is in distinguishing between classes.

## 6. Results and Discussion

We computed precision, recall, F1 score, and AUC for each time-series model trained for 15 and 25 epochs. The models achieved optimal performance at 15 epochs, which we chose as the standard training time for our models. The results of our experiments with 15 epochs are presented in Table 4. A high precision, recall, F1 score, and AUC (close to 1) are desired, as they together indicate a well-fitted model.

As shown in Table 4, the Xception and XCM models achieved the best performance. In particular, Xception and XCM achieved an AUC of 0.96 and F1 score of 0.97. This indicates that the proposed approach based on either Xception or XCM could identify abnormal videos with a high degree of accuracy. Inception achieved average performance with an AUC of 0.77 and F1 score of 0.56, while MiniRocket performed poorly with an AUC of 0.50 and F1 score of 0.46. The results in Table 4 show that the proposed approach based on the combination of feature extraction using bounding boxes, together with time-series models (Xception or XCM), was capable of accurately identifying abnormal videos.

**Figure 10:**
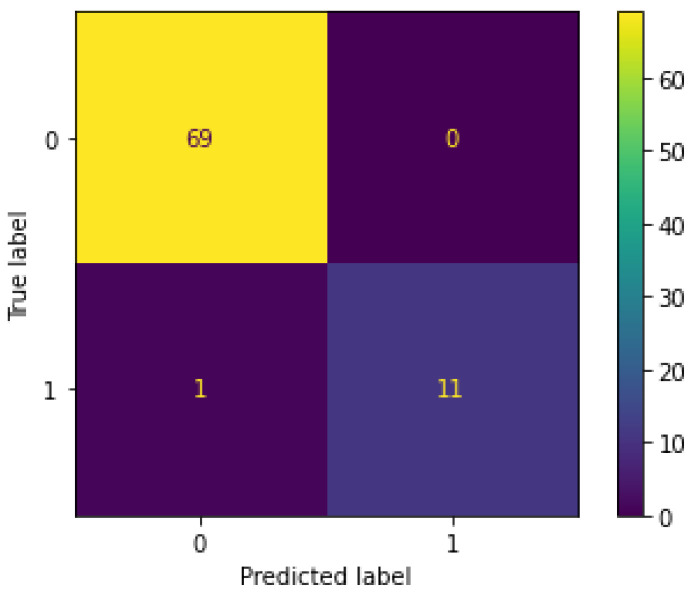
Confusion matrix for the best models.

The confusion matrix for the best models is presented in Figure 10. Neither model misclassified a normal instance as abnormal, and only 1 out of 12 abnormal instances was classified as normal. In other words, the models mistook only 1 instance of shoplifting as regular shopping while accurately identifying the remaining 11 instances of shoplifting.

We further investigate the above misclassification in Figure 10, which was is in Shoplifting Video 34 in the UCF Crime dataset. In particular, this video had over 20,000 rows in the tabular dataset, which were cut down to 15,000. This was particularly odd because the video itself had only 3 people in the entire clip. Furthermore, YOLOv5 with Deep Sort identified about 225 bounding boxes in the video. This was likely what confused the models, since all the other values in the dataset did not have as many people. Hence, this is an issue with the application of YOLOv5 with Deep Sort and the main reason behind the misclassification.

To better compare the tested models, we performed 10-fold cross validation on the dataset. In the context of shoplifting crime prevention, false positives can be costly, as they may lead to unnecessary interventions or the detainment of innocent individuals. Therefore, in this specific application, even a small false-positive rate may render the model totally infeasible in real life. This suggests that precision is more important than recall in this context, as it is crucial to minimize the number of false positives.

Table 4 shows that the highest precision value of 0.99 was achieved by the Xception Time and XCM models, indicating that they are very good at correctly identifying positive samples. This indicates that, out of all the positive predictions by these models, 99% were correct. The precision value for the inception time model was relatively lower, at 0.64, indicating that it had more false positives than the other models did. Considering that even a small false positive rate may render the model totally infeasible in real life, it is clear that the Xception Time and XCM models may be the most appropriate choices for shoplifting crime prevention application, as they achieved the highest precision values. The MiniRocket model, on the other hand, achieved the lowest precision value of 0.43, which may be considered unacceptable in this context.

**Figure 11:**
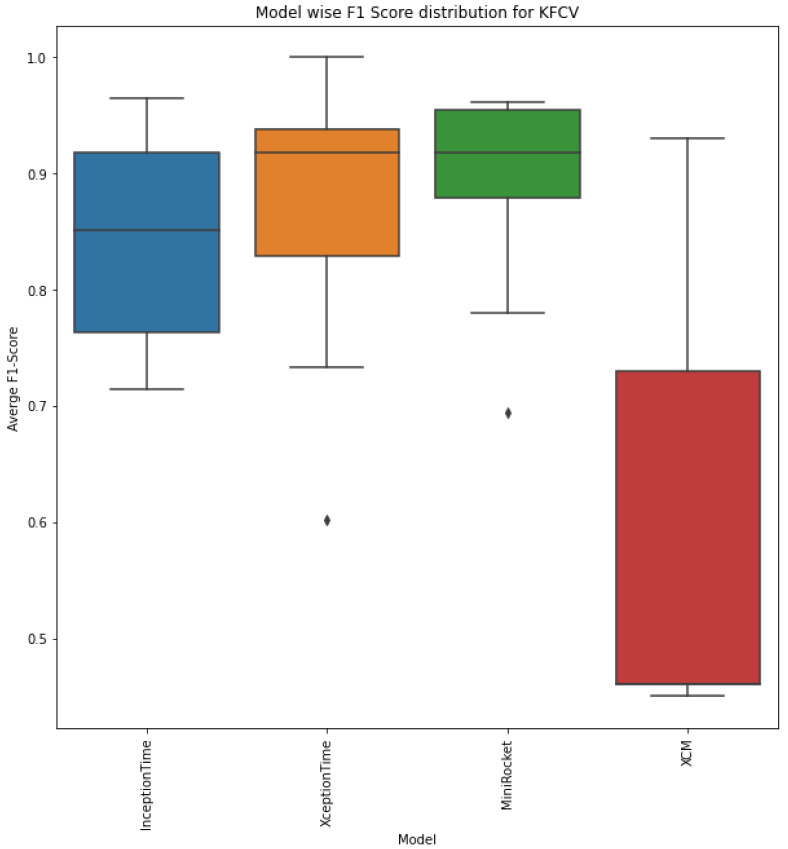
F1 score distributions across 10-fold cross validation for each model.

While recall is also an important metric, the fact that false positives may render the model totally infeasible suggests that precision should be prioritized over recall in this specific application. However, precision and recall are both important metrics, and a balance between the two should be sought in the context of the specific problem. Therefore, the average F1 scores across the 10 folds with the standard deviations and median F1 score for each model are shown in Table 5. Clearly, XceptionTime and MiniRocket performed the best during cross validation, with the highest average F1 scores of 0.87 and 0.89, respectively, and a median F1 score of 0.92. Moreover, XCM actually performed the worst during cross validation, with a mean F1 score of only 0.6 and large variance. The distribution of the F1 scores for each model is better illustrated in the box plot in Figure 11.

Moreover, Xception Time model still achieved the highest precision value with a mean of 0.96 ± 0.04, indicating that it was able to correctly identify positive samples with a high degree of accuracy. The MiniRocket model had the second-highest precision value with a mean of 0.91 ± 0.08, followed by the InceptionTime model, with a mean of 0.86 ± 0.17. The XCM model had the lowest precision value with a mean of 0.63 ± 0.24, indicating that it was less effective at correctly identifying positive samples.

Since a high number of false positives may render the model totally infeasible in real life, the Xception Time model may still be the most appropriate choice for shoplifting crime prevention application, as it achieved the highest precision value in both tables of results. However, the MiniRocket and InceptionTime models also achieved high precision values and may be worth considering.

In terms of recall, the Xception Time model had the highest mean recall value of 0.92 ± 0.06, indicating that it was able to correctly identify a high proportion of positive samples. The InceptionTime and MiniRocket models had slightly lower recall values, with means of 0.85 ± 0.14 and 0.88 ± 0.09, respectively. The XCM model had the lowest recall value with a mean of 0.63 ± 0.19, indicating that it was less effective at identifying positive samples.

The figure shows that MiniRocket was actually the most stable algorithm since its F1 score had the lowest interquartile range, while XCM performed the poorest, with an extremely large interquartile range of about 27%.

Using the nonparametric Kruskal–Wallis test to test for significant differences among the median F1 scores, we obtained a statistic of 11.015 and a *p*-value of 0.012, which led us to reject the null hypothesis (at 5% significance level) that the distributions of all the models had the same median value. Hence, there was at least one model with a statistically significant different median F1 score.

We could use the nonparametric Tukey test to identify whether the median of each distribution of the F1 scores was statistically different, and identify exactly which models had a statistically different F1 score distribution. Table 6 shows the results of the test.

Here, we can clearly see that we were able to reject the null hypothesis (at 5% level of significance) when comparing any model to XCM, but not otherwise. This indicates that XCM had a statistically different distribution to that of the other models, while InceptionTime, XceptionTime, and MiniRocket were comparable. Hence, XCM performed statistically worse than all the other models.

On the basis of the overall distribution of the F1 scores across the 10-fold cross validation, we can conclude that MiniRocket was the best model with a median F1 score of 92% since it had the highest median F1 score and low variance, which rendered it more stable.

We now compare our best models during cross validation, MiniRocket and XceptionTime, to the baseline RTFM [33]. Comparing the baseline model to our median F1 score (92%), we were able to significantly vanquish the given baseline in Table 7.

The imbalance in the dataset classes was easily dealt with by Xception and MiniRocket without requiring any particular preprocessing or data balancing methods such as oversampling. The ability of Xception and MiniRocket to handle imbalanced data without additional preprocessing indicates the robustness of the methods.

A further attractive feature of MiniRocket is that it had a mean F1 score of 89%, which was the same as that for RTFM. This shows that our proposed method was able to perform just as well with a far more intuitive approach to the problem.

**Figure 12:**
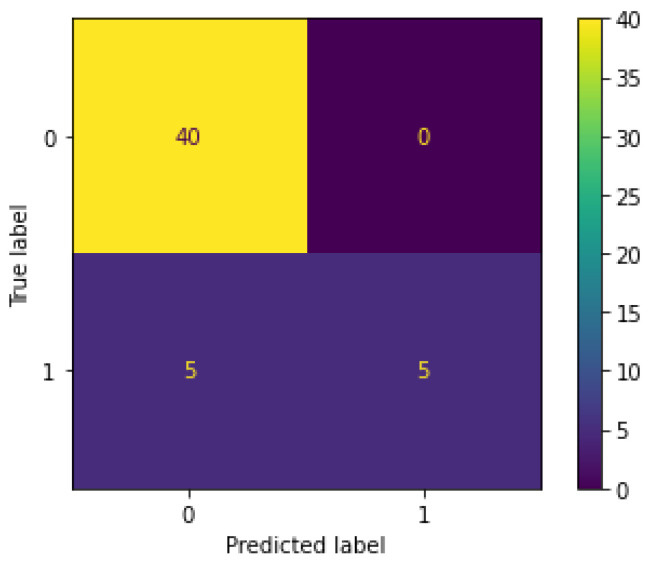
RTFM confusion matrix.

Additionally, as in Figure 12, we notice that RTFM correctly identifies all the normal class instances, but is unable to distinguish between the abnormal instances, with 5 misclassifications and 5 correct classifications. Hence, the RTFM was able to easily learn how normal instances looked like, but became easily confused with abnormal instances.

**Figure 13:**
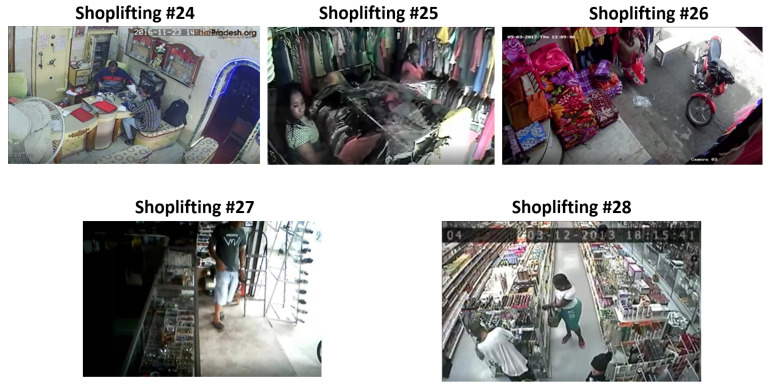
RTFM misclassifications that the proposed method classified correctly.

We could also visually compare the scenes that RTFM misclassified and our approach was able to classify correctly. RTFM misclassified five of the shoplifting scenes (numbers 24 to 28) as normal, which was incorrect. These five incorrect misclassifications can be seen in Figure 13.

By analyzing the videos in Figure 13, one can understand why the RTFM model was unable to detect the misclassifications. A particularly noticeable characteristic of each of the clips that the RTFM failed to classify correctly was that, in all the clips, the subject performing the theft was in the same place for almost the entire clip. Since the RTFM focuses on identifying temporal features from I3D features, we hypothesize that clips with a lack of subject movement lack temporal information for the RTFM to be able to flag them as an anomaly. RTFM aims to capture temporal information between successive frames in a video, but as observed, it is unable to capture anomalies with a lack of movement from the subjects in the clip. The proposed bounding box representation of the videos helps in capturing this temporal information more effectively, and allows for the machine-learning model to better detect anomalies on the basis of the movement of these bounding boxes.

Additionally, the proposed approach outperformed the SOTA regarding inference time. We examined the inference time of the RTFM model and our proposed approach using MiniRocket on a 14 s shoplifting video at 30 frames per second. The prediction was performed 10 times, and the times were averaged to account for any fluctuations in the inference time. The results of the inference test, conducted using an RTX 2060, can be found in Table 8, showing that the proposed approach with MiniRocket outperformed RTFM in inference times by up to 8.45 times faster. The primary reason for this is that RTFM utilizes large amounts of computation time to generate I3D features. On the other hand, the proposed approach provides extremely fast object detection using YOLO with Deep Sort, which leads to much faster preprocessing. Needless to say that the reduction in preprocessing time provides a competitive advantage during inference.

## 7. Conclusions

In this paper, we introduced a new approach to detecting suspicious behavior in videos for crime prevention by utilizing object tracking methods to convert a video classification problem into a time-series classification problem. This use of YOLOv5 with Deep Sort to track people through a video and converting the video data into a tabular time-series format allow for the use of time-series classification models such as Inception Time, Xception Time, XCM, and MiniRocket.

Time-series classification models successfully use the transformed dataset to identify suspicious behavior in videos. The best models were XceptionTime and MiniRocket, achieving the highest median F1 score of 92% during cross validation. These results surpassed the current state-of-the-art model in the field, RTFM, by 3%. Moreover, this method was able to correctly classify some of the clips that the RTFM was unable to classify correctly, showing the robustness of this method. Furthermore, our results demonstrated an impressive 8.45-fold increase in detection inference speed compared to that of the state-of-the-art RTFM.

The success of the paper was in the introduction of a new, more intuitive approach to suspicious behavior detection without the need for data augmentation or image feature extraction. Regardless, the proposed method achieved results similar to or better than those of the current state-of-the-art models in the field. Additionally, the proposed approach is highly generalizable to other problems in anomaly detection in videos by using object tracking. The application of the proposed method to other use cases is an intriguing future research avenue.

## Figures and Tables

**Table 1:** Class distribution after splitting videos into clips.

Dataset Class	No. of Instances
Abnormal	81
Normal	462

**Table 2:** Stratified training and test split of the data.

No. of Instances	Dataset Class	Dataset
12	Abnormal	Testing
69	Normal	Testing
69	Abnormal	Training
393	Normal	Training

**Table 3:** RTFM dataset Split.

No. of Instances	Dataset Class	Dataset
10	Abnormal	Testing
40	Normal	Testing
40	Abnormal	Training
228	Normal	Training

**Table 4:** The performance of the proposed model using different time-series models. Each model was trained for 15 epochs.

Name	Precision	Recall	F1 Score	AUC
Inception Time	0.64	0.77	0.56	0.77
Xception Time	0.99	0.96	0.97	0.96
XCM	0.99	0.96	0.97	0.96
MiniRocket	0.43	0.5	0.46	0.50

**Table 5:** Precision, recall, and F1 scores across 10-fold cross validation. Provided values are mean ± standard deviation.

Model Name	Precision	Recall	F1 Score	Median F1 Score
InceptionTime	0.86 ± 0.17	0.85 ± 0.14	0.84 ± 0.09	0.85
XceptionTime	0.96 ± 0.04	0.92 ± 0.06	0.87 ± 0.13	0.92
MiniRocket	0.91 ± 0.08	0.88 ± 0.09	0.89 ± 0.09	0.92
XCM	0.63 ± 0.24	0.63 ± 0.19	0.6 ± 0.2	0.46

**Table 6:** Results of Tukey’s test.

Group 1	Group 2	Mean Difference	*p*-Value	Lower	Upper	Reject
InceptionTime	MiniRocket	0.0446	0.8783	−0.1167	0.2059	False
InceptionTime	XCM	−0.2413	0.0015	−0.4026	−0.08	True
InceptionTime	XceptionTime	0.0259	0.9725	−0.1354	0.1872	False
MiniRocket	XCM	−0.2859	0.0002	−0.4472	−0.1246	True
MiniRocket	XceptionTime	−0.0187	0.9892	−0.18	0.1426	False
XCM	XceptionTime	0.2672	0.0004	0.1059	0.4284	True

**Table 7:** Baseline comparison.

Model Name	Feature Type	F1 Score
RTFM	I3D RGB	0.89
Ours–XceptionTime	Custom	0.92
Ours–MiniRocket	Custom	0.92

**Table 8:** Inference times.

Model Name	Preprocessing Time (s)	Total Inference Time (s)
RTFM	13.7	13.9
Ours–MiniRocket	1.5	1.6

## Data Availability

The data used in this study are publicly available online [27].
